# A Plea for Neutral Comparison Studies in Computational Sciences

**DOI:** 10.1371/journal.pone.0061562

**Published:** 2013-04-24

**Authors:** Anne-Laure Boulesteix, Sabine Lauer, Manuel J. A. Eugster

**Affiliations:** 1 Department of Medical Informatics, Biometry and Epidemiology, Ludwig-Maximilians-University of Munich, Munich, Germany; 2 Department of Statistics, Ludwig-Maximilians-University of Munich, Munich, Germany; 3 Helsinki Institute for Information Technology, Department of Information and Computer Science, Aalto University, Espoo, Finland; Politecnico di Torino, Italy

## Abstract

In computational science literature including, e.g., bioinformatics, computational statistics or machine learning, most published articles are devoted to the development of “new methods”, while comparison studies are generally appreciated by readers but surprisingly given poor consideration by many journals. This paper stresses the importance of neutral comparison studies for the objective evaluation of existing methods and the establishment of standards by drawing parallels with clinical research. The goal of the paper is twofold. Firstly, we present a survey of recent computational papers on supervised classification published in seven high-ranking computational science journals. The aim is to provide an up-to-date picture of current scientific practice with respect to the comparison of methods in both articles presenting new methods and articles focusing on the comparison study itself. Secondly, based on the results of our survey we critically discuss the necessity, impact and limitations of neutral comparison studies in computational sciences. We define three reasonable criteria a comparison study has to fulfill in order to be considered as neutral, and explicate general considerations on the individual components of a “tidy neutral comparison study”. R codes for completely replicating our statistical analyses and figures are available from the companion website http://www.ibe.med.uni-muenchen.de/organisation/mitarbeiter/020_professuren/boulesteix/plea2013.

## Introduction

The main goal of methodological research in computational sciences (including, e.g. bioinformatics, machine learning, or computational statistics) is the development of new methods. By development of new methods, we mean that the researchers suggest new procedures for analyzing data sets. The new procedure should be applicable to specific substantive research questions, but these substantive research questions are (often) not the primary center of interest of the methodological researcher. New methods are expected to “make the world better” by, roughly speaking, making the results of statistical analyses closer to the truth. Surprisingly, comparison studies and reviews investigating the closeness to the truth are sometimes considered as less exciting and less useful by many researchers or by most journal editors, and often implicitly excluded from the journals’ scopes.

This is in strong contrast to clinical research. The ultimate goal in clinical research is to make the world better by somehow improving the health outcome of patients (or/and reducing the cost while maintaining the same outcome), for instance through a specific drug, therapy or prevention strategy. Roughly speaking, the clinical analogue of a computational article suggesting a new method would be an article suggesting a new intervention for improving health outcome. Yet, most published medical papers do not directly suggest such a new measure. Many other types of clinical research projects are conducted, for instance large validation studies, phase IV clinical trials, or meta-analyses. Of course, crucial differences between computational science research and medical research make comparisons only partially pertinent. Research on algorithms and methods does not follow the same rules as research involving human beings with direct potentially vital consequences. The development of a new drug or new prevention strategy essentially requires more time, money, caution and coordination than the development of a new statistical method. Some principles, however, hold for both worlds. If we focus on the problem of comparison studies considered in this paper, the question is whether we can imagine a world in which clinical journals accept to publish only underpowered phase I or II clinical trials evaluating new therapies but no phase III or IV trials. The answer is of course no. In data analysis journals, however, the equivalent of phase III and IV trials, i.e. well-planned and well-conducted comparison studies in our metaphor, are often considered as not deserving publication. Note that the importance of comparison studies and their lack of consideration in the literature is not specific to computational science and has been recognized in other fields such as experimental biosciences [1].

We claim that comparison studies in computational sciences may be necessary to ensure that previously proposed methods work as expected in various situations and that emerging standard practice rules adopted by substantive researchers or statistical consultants are the result of well-designed studies performed by computational science experts. The community tends to establish standards and guidelines as time goes by. In an ideal world, these standards are the results of well-done comparative studies and consensus from independent teams. However, other factors might contribute to promote a particular method, including the reputation of the authors or the impact factor of the journals the method was published in. From the point of view of applicants (say, for example, biologists), further criteria include the availability of well-documented and user-friendly implementations of the method or an application of this method in one of the few leading scientific journals that other scientists tend to imitate. These quantitative objective criteria may seem natural. After all, a method published by a renown author in an excellent journal is more likely to work well than a method published by an unknown author in a low-ranking journal. Availability of good software is of course a crucial advantage for applicants who would not be able or would not have the time to implement any of the methods themselves. And a method that worked well in a previous well-published study is perhaps more likely to also work well in future studies than another method.

It is unclear, however, whether standard practice rules should be established solely on such subjective criteria. Would it not be better to give more importance to comparison studies? One may of course argue that comparison studies can be performed within original articles presenting new methods. Indeed, in practice new methods are usually compared to a few existing methods in order to establish their superiority. Such comparison studies are extremely important for illustrative purposes, i.e. to demonstrate that the developed method is applicable in practice and yields acceptable results, but should strictly speaking not be considered as comparison studies because they are often substantially biased and thus not *neutral*.

For example, in the context of clinical outcome prediction or diagnosis based on high-dimensional “omics” data (such as, e.g. microarray gene expression data), hundreds of articles presenting new supervised classification algorithms have been published in the bioinformatics, statistics and machine learning literature. Almost all of them claim that the new method “performs better” than existing methods. Most often these claims are based on small real data studies including a few exemplary data sets. The fact that for more than ten years hundreds of authors have been claiming that their new method for classification using microarray data outperforms existing ones suggests that something goes wrong in the comparison studies performed in these articles. Similar discussions can be found in other fields of application of machine learning and computational statistics [2].

In this paper we present a survey of recent computational papers on supervised classification published from 2010 to 2012 in seven high-ranking journals in the fields of bioinformatics, computational statistics and machine learning. The goal of our survey is to provide an up-to-date picture of current scientific practice with respect to the comparison of methods in both articles presenting new methods and articles focusing on the comparison study itself. To keep the survey feasible, the focus is set on supervised classification, a topic within our own area of expertise that is highly relevant in bioinformatics, computational statistics and machine learning.

We then take the results and insights given by the survey as a starting point to critically discuss the necessity, impact and limitations of neutral comparison studies in computational sciences. In particular, we define three reasonable criteria a comparison study has to fulfill to be considered as neutral. Furthermore, we explicate general considerations on the individual components of a “tidy neutral comparison study” and argue for the publication of negative results and pitfalls. We consequently draw parallels to clinical research and clinical studies in order to motivate and illustrate our statements, decisions and arguments.

## Methods

**Figure 1:**
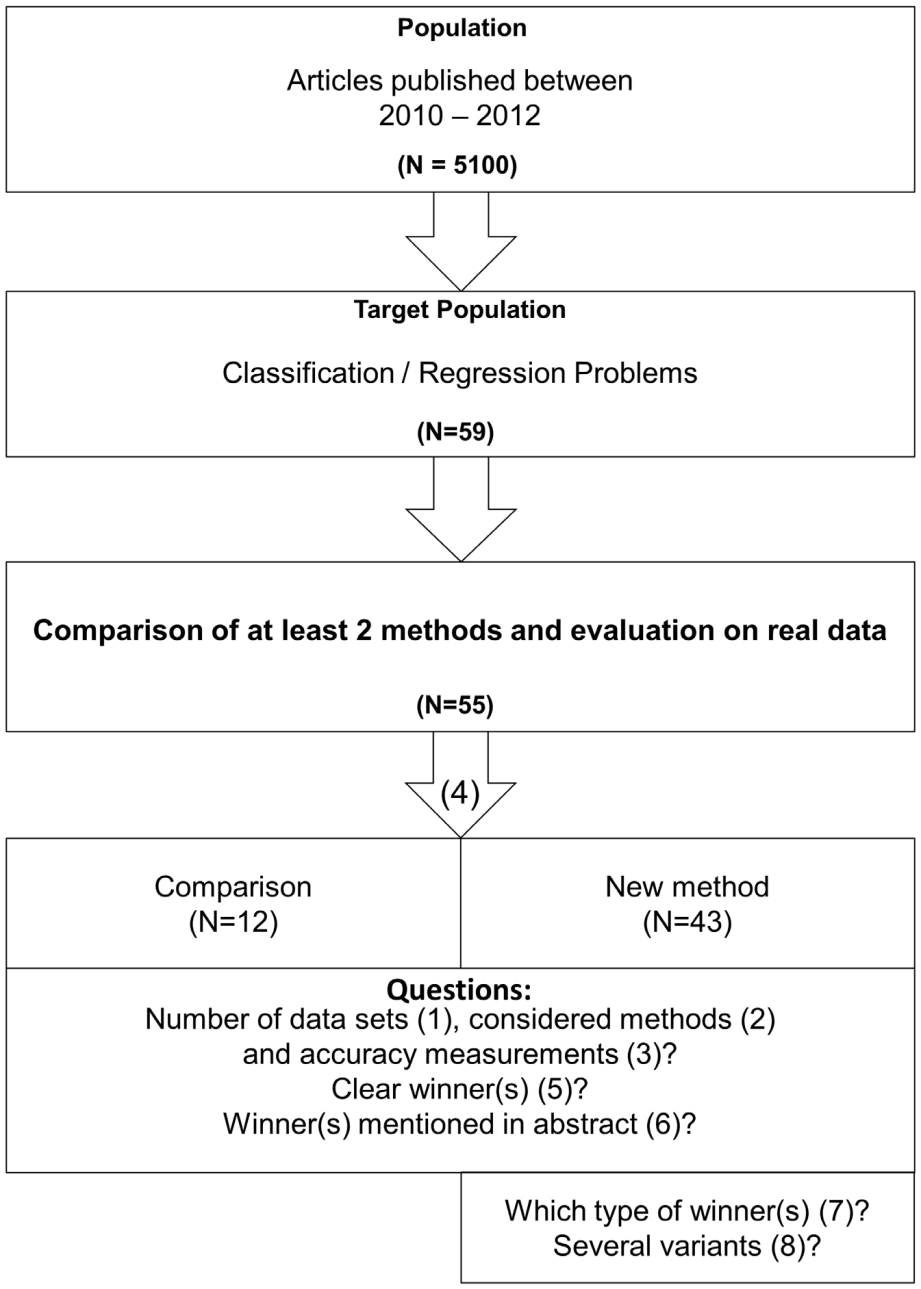
Flowchart of the paper selection process and recorded features. The numbers in parentheses refer to the numbering of the recorded features defined in the Methods section.

We designed a study to provide an up-to-date quantitative picture of real data comparison studies presented as part of published papers with emphasis on neutrality issues and whether the studies identify winners. Figure 1 visualizes the article selection process and recording of comparison study features.

### Research Fields, Journals and Articles

Given the large number of research fields related to computational science, we decided to focus on three fields in the area of our expertise and research interests: bioinformatics (BI), computational statistics (CS) and machine learning (ML). We chose seven representative high-ranking journals (Table 1 lists them), and restricted the survey to articles published in these journals between 2010 and 2012. We also decided to focus on a particular topic, namely on supervised classification as an important topic within computational science.

**Table 1:** Overview: Published articles dealing with supervised classification problems in the period from 2010 to 2012.

Journal	# Articles
**Bioinformatics (BI)**	**16**
*Bioinformatics (until Volume 28 Issue 23, 2012)*	6
*BMC Bioinformatics (until Volume 13– October 15, 2012)*	10
**Computational Statistics (CS)**	**17**
*Computational Statistics (until Volume 27, Issue 4, 2012)*	4
*Computational Statistics & Data Analysis (until Volume 56, Issue 12, 2012)*	11
*Journal of Computational and Graphical Statistics (until Volume 21, Issue 3, 2012)*	2
**Machine Learning (ML)**	**26**
*Journal of Machine Learning Research (until Volume 13, 2012)*	15
*Machine Learning (until Volume 89, Issue 1–2, 2012)*	11
**Total**	**59**

Obviously, these choices are not random, and consequently neither the journals nor the papers are random samples. These limitations of our study design were necessary (i) to keep the study practically manageable and to allow us to spend the required time on each of the papers, (ii) to ensure that the selected papers are homogeneous enough to be adequately compared, and (iii) to avoid misinterpretations that would inevitably occur for papers outside our field of expertise. However, we want to stress that at any rate, we neither “tuned” the set of journals during the study, nor did we choose them because we expected them to yield a particular results pattern.

The defined survey setting results in a total set of 5100 articles. We then conducted a manual screening of the articles based on their titles to select those dealing with supervised classification, i.e., from a statistical point of view, regression for categorical dependent variables. A manual screening was preferred to a systematic database search based on keywords, because we realized in a small pilot study that many articles do not mention supervised learning or classification as keywords or in the title but, for example, rather the name of the specific learning method which is considered.

### Inclusion Criteria

The goal of the study is to explore common scientific practice in comparing supervised classification methods. Therefore, for each article related to supervised classification we first report.

1. whether the article presents a comparison study comparing two or more methods.

Comparison studies can be executed on simulated/artificial data or so-called “real world” data. This could make a difference for the inference of “standard practice rules”, since real world data are known to often behave differently from simulated data; therefore, we report.

2. whether the article includes an evaluation on real data.

Since one of our main claims in the paper is that comparison studies are important to infer “standard practice rules” we focus the survey on articles fulfilling both Condition 1 and Condition 2; therefore, we report for each article.

3. whether the article includes an evaluation of two or more methods on real data.

### Comparison Study Features

We designed the following eight observable comparison study features to capture information on common practice in the context of comparison studies based on real data. To address the issue of neutrality (which is essentially a latent unobservable variable), we particularly focused on the differentiation between comparison studies published as part of a paper introducing a new method and comparison studies on existing methods whose contribution is the comparison itself.

#### Basic features

For each comparison study we recorded the following basic features:

1. Number of considered real data sets
2. Number of considered methods/variants
3. Number of accuracy measures

#### Purpose of a comparison study

Comparison studies can be executed for two different purposes: to illustrate a newly developed method and compare it against established competing methods, or to compare existing methods in a more “symmetric approach” in order to provide recommendations for substantive researchers who want to analyse the data they produced. Therefore, we report.

4. whether the purpose of the comparison study is to introduce a new method or to compare existing ones;

#### Reported main results

Based on the conclusion drawn by the authors (i.e. without looking ourselves at the figures reported in the paper), we report.

5. whether the comparison study identifies one or several methods as (a) clear winner(s) according to the conclusion section, and if yes,

6. whether the winners, if any, are mentioned in the abstract;

#### Introduction of a new method

If the comparison study focuses on the introduction of a new method, we report.

7. whether the new method belongs to the identified winners (the answer “no” would imply a “negative result”) and if yes, which type of winner(s) it is (see the definition of winner categories in the Results section);

8. whether there are several variants of the method presented.

### Data Analysis

The collected features were examined using descriptive statistics and exploratory data analysis methods. The goal of the data analysis was to quantitatively describe the common practice in the context of comparison studies based on real data. Note that no inferential statistical methods (e.g., statistical significance tests) were used. The limited sample size and the potential dependence between papers (published in the same journal or, e.g. by the same team) make the validity of standard statistical inference methods questionable.

## Results

In this section we present some results of the survey. All results can be reproduced using R-codes and data available from the companion website http://www.ibe.med.uni-muenchen.de/organisation/mitarbeiter/020_professuren/boulesteix/plea2013, following recommendations for computational reproducibility of research papers [3].

### Articles

The initial situation was a population of 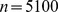 articles published in the three computational science fields bioinformatics (BI), computational statistics (CS) and machine learning (ML).

The manual screening identified 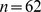 articles based on their titles. The word “classification” or “classifier” was found in 40 of these titles, while 24 titles included the name of a specific method. The words “comparison” and “comparative” were found in four titles. The word “prediction” also appeared in four titles, while the words “learning” and “discrimination” were found in five titles and two titles, respectively. Three papers were excluded after further consideration because they did not fit our definition of supervised classification in spite of their title, finally yielding a total of 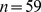 papers. Their references can be found in File S1.

**Figure 2:**
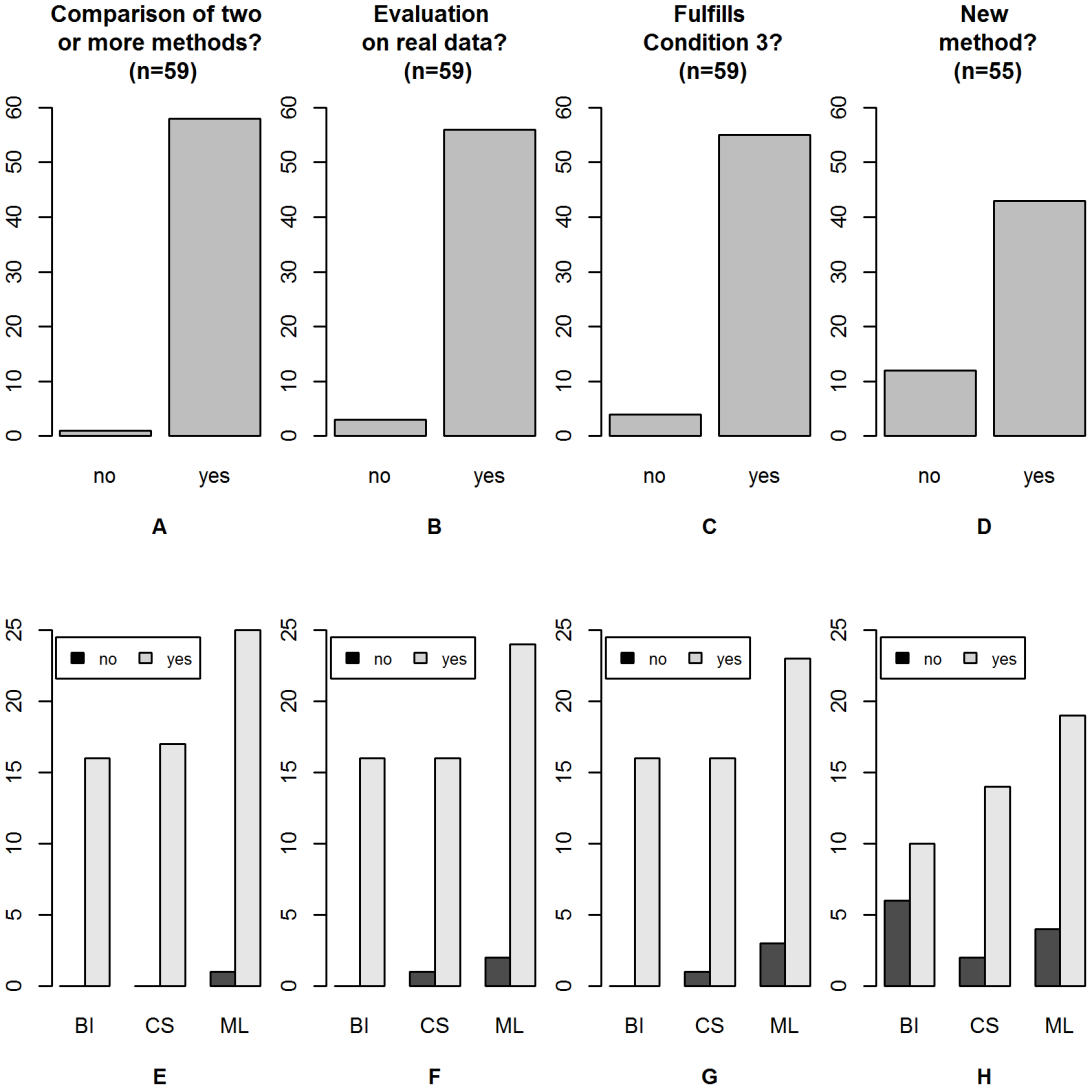
Overview of selected papers and selection criteria. The top panel shows the global results for all papers relevant to the respective question (either all 59 papers or the 55 papers satisfying Condition 3), while the bottom panel shows the results stratified by research field (bioinformatics: BI, computational statistics: CS and machine learning: ML). (A) and (E): Does the paper include a comparison of two or more methods?; (B) and (F): Does the paper include an evaluation on real data?; (C) and (G): Does the paper fulfill Condition 3?; (D) and (H): Does the paper present a new method?

Out of these 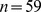 articles, 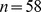 articles presented a comparison of two or more methods as displayed in Figure 2A and Figure 2E. Three out of 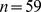 articles did not present any comparison study based on real data, as displayed in Figure 2B and Figure 2F. This resulted in a total of 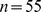 articles satisfying Condition 3, on which we focused in the rest of our survey; see Figure 2C and Figure 2G.

Out of these 55 articles, 43 articles presented new methods and 12 articles presented comparison studies of existing methods only, as shown in Figure 2D and Figure 2H.

### Basic Comparison Study Features

Each of the 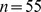 articles fulfilling Condition 3 presented a comparison study of at least two methods based on real data. We recorded the number of data sets, number of included methods and number of accuracy measurements considered in each paper.

**Figure 3:**
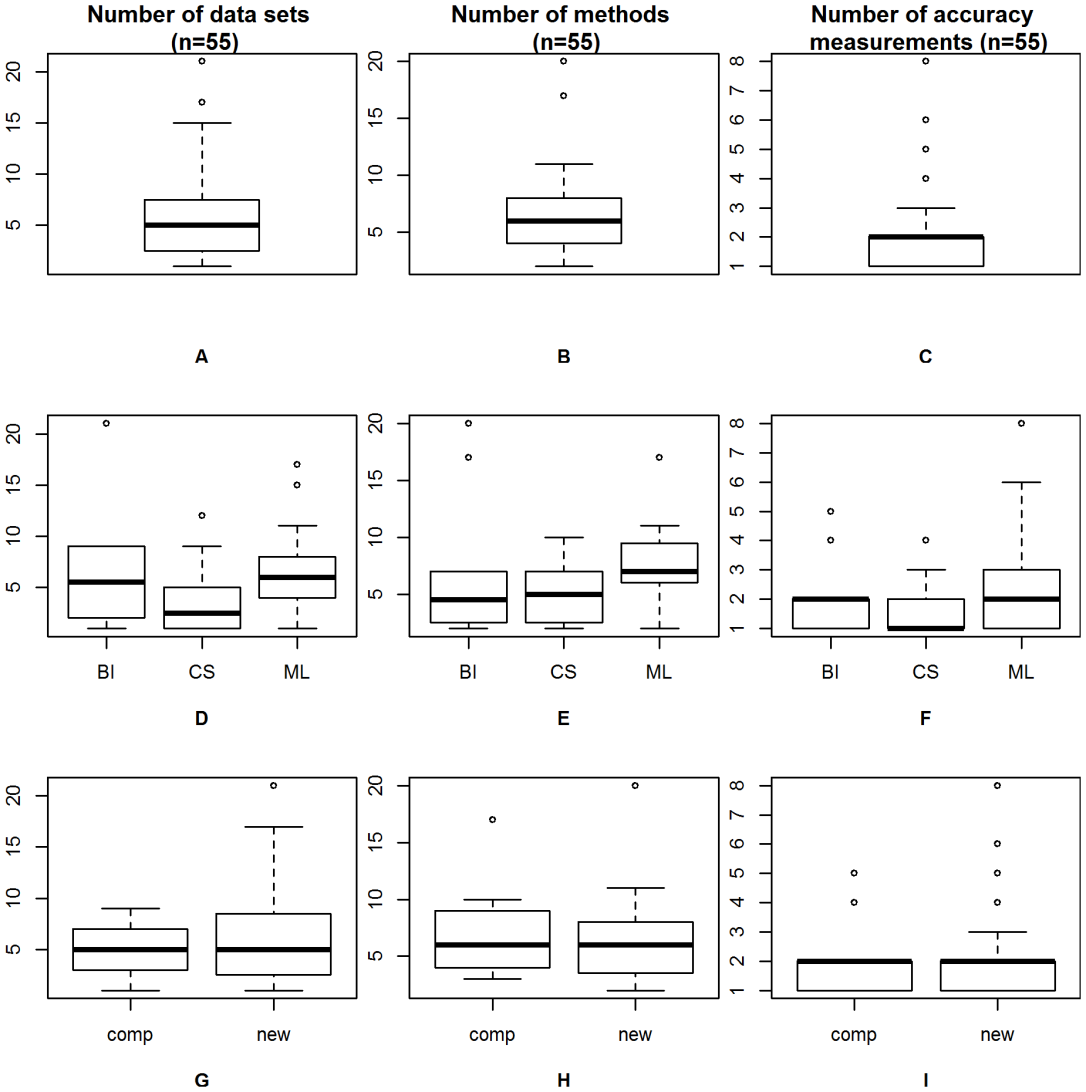
Number of data sets, methods and accuracy measurements. The top panel depicts the results for all 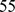 papers, the results in the middle panel are stratified by research field (bioinformatics: BI, computational statistics: CS and machine learning: ML), and the bottom panel differentiates between “comparison” and “new method” papers. (A), (D) and (G) show the number of data sets, (B), (E) and (H) the number of methods, (C), (F) and (I) the number of accuracy measurements.

As can be seen from Figure 3A, the median number of data sets considered in the comparison studies was 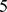, while the mean was 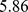. When looking at the boxplots separately per research field, see Figure 3D, the highest mean was found in the machine learning articles with 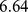, while the medians varied from 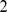 in computational statistics to 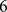 in machine learning. The maximal number of 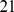 data sets was found in a bioinformatics journal.

Figure 3B depicts the number of methods included in the comparisons. The median was 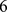, while the mean was 6.36. A separate examination (Figure 3E) shows the highest median and mean for the machine learning papers with a median of 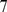 and a mean of 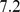 included methods. The maximum of 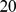 included methods was again found in a bioinformatics journal.

Finally, the mean number of accuracy measurements was 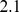 and the median was 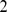 as depicted in Figure 3C, while the maximum of 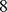 accuracy measurements was found in the machine learning articles (Figure 3F).

For illustrative purposes these basic features are also separately shown for the papers presenting a new method and for papers focusing on the comparison itself. These boxplots (Figure 3G, Figure 3H, Figure 3I) show that the number of data sets, number of methods and number of accuracy measures were not substantially different for comparison studies and articles presenting new methods.

### New Methods and Clear Winners

A more delicate question is whether in comparison studies which introduce a new method clear winner(s) among the considered methods are reported in the conclusion. Having the so called “no-free lunch theorem” [4] in mind, we do not expect papers claiming that one of the methods, for instance the new method, outperforms all competitors for all classification problems.

We identified three main profiles: (W1) comparison studies claiming that the new method outperforms the other methods in general with respect to the chosen accuracy measure; (W2) comparison studies arguing that the new method shows performances similar to existing methods in terms of accuracy but has either other important advantages (for instance computational efficiency) or better performance in some specific cases that makes it a serious competitor; and (W3) comparison studies just saying that the new method performs similarly to other methods without pointing to a specific advantage.

Note that the concept of winner and the classification of the papers into one of the categories W1, W2, W3 implies a high level of subjectivity, since the definitions are intentionally kept vague. In contrast to biomedical research where, say, the 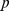-value of the association between outcome and prognosis factor is either significant or non-significant, there are to date no well-established or/and natural criteria to classify the results of methodological computational papers. To make our classification more transparent, we give examples of typical statements for each of the three categories in Table 2. This definition problem underlines the importance of reporting issues in computational literature–a topic that has barely focused any attention so far but might be further investigated in future research.

**Table 2:** Examples for the three winner categories.

Category	Examples
W1	superior
	“always leads to improved prediction performance”
	“the proposed method efficiently reduces prediction errors”
	“the proposed algorithm is superior to existing methods”
W2	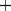 competitive
	“has the additional computational advantage”
	“a more cost-efficient classifier that is at least as good, and sometimes better”
	“reduction in computational time for training the algorithm”
W3	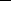 competitive
	“the drawback of our method is that”
	“can be more expensive to compute”
	“further research might be needed”

**Figure 4:**
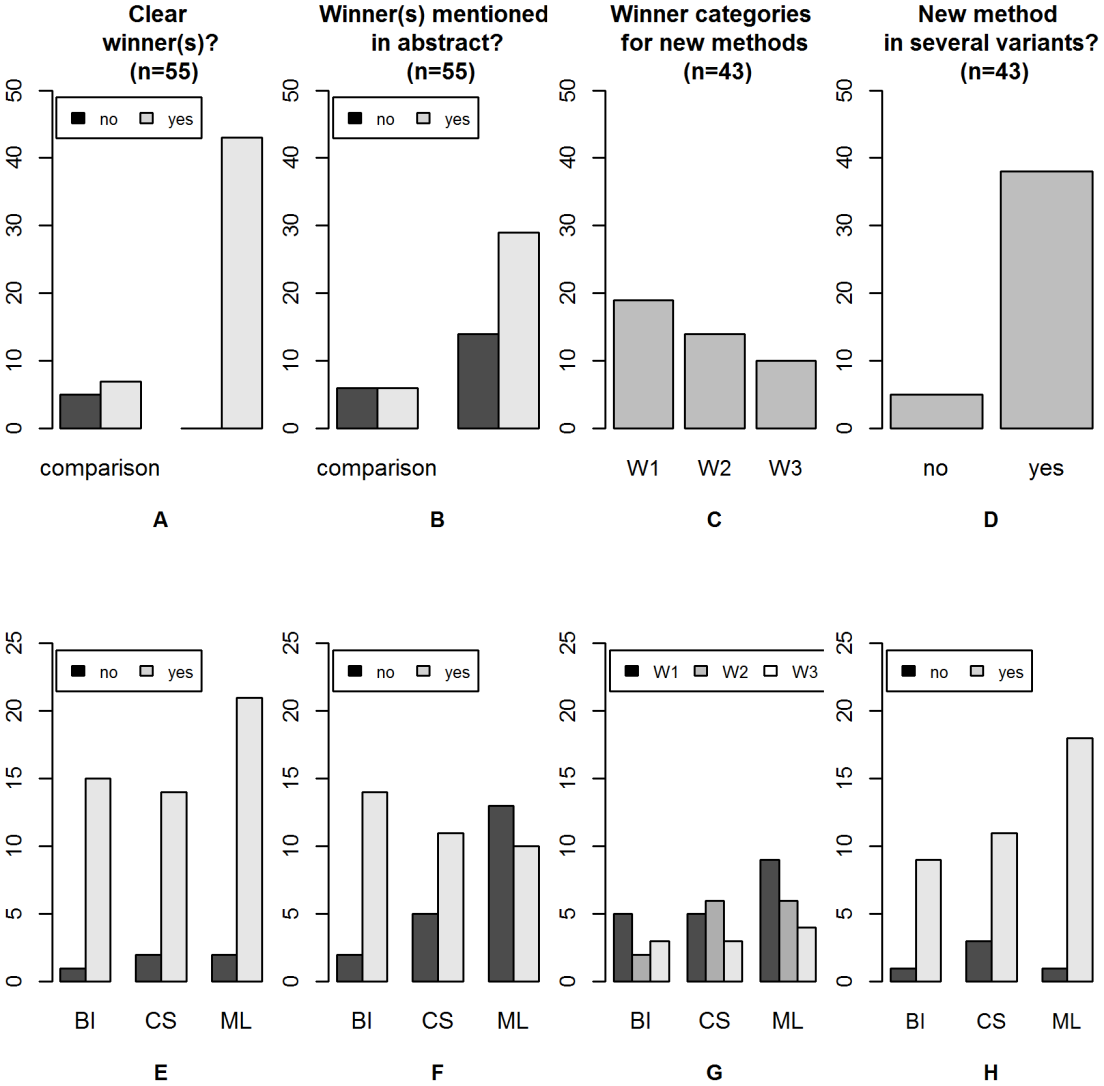
Reporting of winners. The top panel shows the global results for all papers relevant to the respective question (either all 55 papers satisfying Condition 3 or the 43 papers on new methods), while the bottom panel shows the results stratified by research field (bioinformatics: BI, computational statistics: CS and machine learning: ML). (A) and (E): Are there clear winner(s)?; (B) and (F): Are the winner(s) mentioned in the abstract?; (C) and (G): Which type of winner(s) (W1, W2, or W3) is identified? (for the 43 papers on new methods only); (D) and (H): Is the new method presented in several variants? (for the 43 paper on new methods only).

Five out of 12 comparison studies did not identify any clear winner(s) (see Figure 4A). In contrast, all 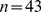 papers presenting new methods belonged to one of these three categories W1, W2 or W3. Within these 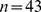 papers, all three categories (W1, W2, W3) had similar frequencies, with a slight advantage for W1 (see Figure 4C). The frequencies of the three categories were not substantially different in bioinformatics, computational statistics and machine learning journals (see Figure 4 G). The winner(s) was (were) often mentioned in the abstract, especially in bioinformatics (see Figure 4B and 4F). In most papers on new methods, several variants of the new method were considered (see Figure 4D and 4H).

As expected, no negative results were published, i.e., no paper suggested a new method that finally turned out to perform worse than existing methods.

## Discussion

In our survey based on 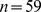 papers recently published in computational journals, we observed that i) comparison studies published as part of papers presenting new methods (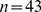) very often identify the new method(s) as (a) winner(s), while pure comparison studies (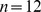) do not always identify winners, ii) computational journals publish much less pure comparison studies than new method papers, iii) papers suggesting new methods are never negative with respect to the new method.

### Limitations

Our study is limited to a set of seven selected journals and to a specific data analysis topic (supervised classification). This possibly implies a selection bias, in the sense that considering other journals and/or other research topics may lead to different results.

Such a selection bias cannot be ruled out–even though the chosen journals do not seem to have an unusual publication policy regarding the investigated issues and the chosen topic is not known to follow an untypical pattern as far as the considered questions are concerned. Our selection of papers was not random since we focused on specific journals, but it was random with respect to the main outcome in the sense that we did not consider any information or “prejudice” concerning the investigated aspects when choosing the journals. The selected papers were also not random with respect to the topic since the topic was fixed from the beginning. Our results and conclusions are thus limited to this topic, although we expect that many aspects are generalizable to other topics. This could be investigated in future research.

Most importantly, we stress that a study involving all computational journals and all topics within computational science would pose numerous methodological problems as well. Beyond the fact that such a large study would hardly be manageable from a practical point of view, the definition of common criteria for the evaluation of papers on completely different research problems or the coordination of efforts among cooperation partners from different expertise areas would be arduous challenges, to cite only a few. In the field of computational sciences literature surveys are still in their infancy, with no well-established procedures and methodologies as it is the case in biomedicine, hence the need to focus on a specific manageable issue in our study. In the long term, however, more effort could be spent on the methodological aspects of surveys in computational sciences. We believe that such efforts are needed and could be highly beneficial to the computational science community.

In the rest of this section, we further reflect on these three striking results by focusing on over-optimism issues and the need for neutral comparison studies, by defining what we consider as a tidy neutral comparison study, and by discussing the issue of negative findings.

### Over-optimism and the Need for Neutral Comparison Studies

Comparison studies included in original research articles presenting new methods might often be over-optimistic with respect to the superiority of the new method, as confirmed by our survey that shows that new methods are often reported to be better than existing methods in some sense, but never worse. Some reasons for over-optimism are empirically assessed in detail and discussed elsewhere [5], [6] in the context of supervised classification using high-dimensional molecular data. The first and perhaps most obvious reason for over-optimism is that researchers sometimes “randomly search” for a specific data set such that their new method works better than existing approaches, yielding a so-called data set bias already investigated in the literature [7]. A second source of over-optimism, which is related to the optimal choice of the data set mentioned above, is the optimal choice of a particular setting in which the superiority of the new algorithm is more pronounced. For example, researchers could report the results obtained after a particular feature filtering which favors the new algorithm compared to existing approaches. The third and probably most subtle problem is that researchers often tend to optimize their new algorithms to the data sets they consider during the development phase [5], [6]. This mechanism essentially affects all research fields related to data analysis such as statistics, machine learning, or bioinformatics. Indeed, the trial-and-error process constitutes an important component of data analysis research. As most inventive ideas have to be improved sequentially before reaching an acceptable maturity, the development of a new method is per se an unpredictable search process. The problem is that this search process leads to an artificial optimization of the method’s characteristics to the considered data sets, as empirically demonstrated in a study on supervised classification [5]. This optimization process over different variants of the new method probably often partly takes place before publication and thus remains confidential. It is common practice, however, to report the performance of several variants in the final paper, as clearly shown by our survey. In both cases, the superiority of the novel method over an existing method (for instance as measured through the difference between the cross-validation error rates) might be considerably overestimated through this optimization process.

Other reasons are of technical nature and related to the ability of the researchers to use the compared methods properly. For example, if an implementation problem occurs with the competing approaches and slightly worsens their results, researchers often tend to spontaneously accept these inferior results. Conversely, they would probably obstinately look for the programming error if such problems occur with their new algorithm. In the same vein, they may unintentionally set the parameters of competing methods to sub-optimal values, or choose a variant of the method that is known by experts to be sub-optimal. They may also select competing methods in a sub-optimal way, i.e. consciously or subconsciously exclude the best methods from the comparison for any reason. Beyond the problems of technical expertise and optimization bias, interpretation and representation issues might also affect the final conclusions of a comparison study. Given the same quantitative outputs, the impression of the reader can be affected by the choice of the vocabulary in the results section, by graphical representation, or by the choice of the main quantitative criterion used to compare the methods. For all these reasons, many comparison studies published in the literature as part of an original paper are substantially biased. In an ideal world, scientific practice might be improved to reduce the impact of the optimization problems discussed above. It is questionable, however, whether these problems can all be fully ruled out in the medium term–even if most researchers show good will to address these issues.

Based on these thoughts, we stress the importance of neutral comparison studies that we define as follows:

A. The main focus of the article is the comparison itself. It implies that the primary goal of the article is not to introduce a new promising method.

B. The authors should be reasonably neutral. For example, an author who has published an article on a new method six months before is likely to be less neutral than an author who has often used several of the considered methods for statistical consulting and, say, previously investigated three of them more precisely in methodological articles. Although an informal check based on the authors’ publication lists found on their homepage suggested that it was not a problematic issue for the 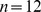 comparison studies included in our survey, non-neutrality of the authors may induce a bias in general.

C. The evaluation criteria, methods, and data sets should be chosen in a rational way, see Section 4 for a more extensive discussion of this problem.

In this perspective, comparison studies presented as part of papers on new methods should be considered as valuable illustrations but, strictly speaking, not as comparison studies. Going one step further, one could say that the authors of a new method should present a convincing illustration that would typically suggest that a method is promising, but leave the actual neutral comparison to other teams in a collaborative context.

Note that in papers on new methods the comparison between the competing methods is essentially not affected by the bias discussed in the previous section. Hence, an idea could be to extract neutral comparisons from comparison studies included in original articles presenting new methods–by considering the competing methods only. However, one should keep in mind that these methods probably have not been given as much attention as in the case of a real neutral comparison study that does not involve any new method. This relative lack of attention possibly leads the underestimation of their performance. In our survey, the number of methods considered in the comparisons was not substantially lower for the 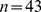 papers focusing on new methods than for the 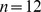 comparison studies. This suggests that in the former papers less attention is devoted to each compared method, since the authors spend a lot of time on the development of the new method.

To come to the point, in an original article on a new method, the focus is on the new method, and that is where the authors generally spend most of their energy. Consequently, comparisons between competing methods should not be over-interpreted because they may be of sub-optimal quality. On this account we make a (passionate) plea for neutral comparison studies in computational sciences.

### Tidy Neutral Comparison Studies

In the same way clinical research and clinical studies have to be well planned and executed (following strict guidelines), comparison studies should also follow a well-defined study design. They should be based on a sound theoretical framework, appropriate analysis methods, and carefully selected components. There is a variety of literature on the design and analysis of comparison studies available. In the context of supervised learning we propagate, for example, Hothorn et al [8] as a theoretical framework and Eugster et al [9] as its practical implementation. However, regardless of the concrete framework, general considerations on the individual components–evaluation criteria, methods and method parameters, and data sets–can be made.

#### Choice of evaluation criteria

In the case of supervised classification algorithms, simple evaluation criteria are, among others, the error rate or preferably the area under curve that is based on the predicted class probabilities. Such criteria are natural and objective. Our survey showed that most published comparison studies consider very few of these criteria, which can be seen as a weakness. However, many other criteria have an impact on the usefulness of a method in practice for applications. From a pedagogical point of view, one should not forget that the method is destined to be used by experts or non-expert users. Therefore, all other things being equal, simplicity of a method constitutes an important advantage, similarly to the clinical context where the simplicity of a therapy protocol should be seen as a major advantage. From a technical point of view, particular attention may be devoted to computational aspects such as computation time and storage requirements (similarly to the costs in the clinical context), the influence on initial values in an iterative algorithm, or more generally the dependence on a random generator (similarly to the robustness of the therapy’s effect against technical problems or human errors).

#### Choice of methods and method parameters

The choice of methods is a very subjective one. At any rate, the concrete choice should be clearly motivated and personal preferences and similar influences should be clearly acknowledged. Researchers are inevitably conducted by personal preferences, past experiences and own technical competence, even if this aspect was not clearly apparent in the 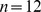 comparison studies of our survey. However, the choice should also be guided by objective arguments. Possible criteria are i) the popularity of the methods in practice (for instance: restrict to methods that have been used in at least three concrete studies), ii) results available from the literature (for example from a previous comparison study) to pre-filter good candidates, or iii) specific pre-defined criteria specifying the nature of the method, for example “only statistical regression-based methods”. None of these criteria should be considered as mandatory for a neutral comparison study. But we claim that, the set of criteria being defined, the methods should be more or less randomly sampled within the range of available methods. As far as method parameters like hyperparameters are concerned, they should be chosen based on “standard practice rules”.

#### Choice of data sets

Researchers performing comparison studies also choose data sets. Considering the high variability of relative performance of methods across data sets and the moderate number of data sets considered in each study (median number of 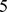 in our survey), a comparison study based on different data sets may obviously yield substantially different results. In the context of supervised classification chosen as an example in our paper, variability arises both because error estimation with standard resampling-based estimators is highly variable for a given underlying joint distribution of predictors and response [10] and because different data sets also have different underlying distributions. Therefore, it is important to make a selection of data sets that is “as representative as possible” to cover the domain of interest. At best, the data sets are chosen from a set of data sets representing the domain of interest using standard sampling methodology.

In summary, many choices have to be met when performing a comparison study, for example, in the case of supervised classification with high-dimensional data: the included methods (e.g. penalized regression, tree ensembles, support vector machines, partial least squares dimension reduction, etc), the considered variants (which kernel for SVM, which fitting algorithm for penalized regression, which optimality criterion for PLS, which splitting criterion for tree ensembles, etc), the data domain (which type of data sets), the parameter tuning procedure (which resampling scheme, which candidate values). With this in mind, it is clear that the topic of interest cannot be handled completely by a single comparison study. Different comparison studies with similar scope may yield different conclusions. This can be seen as a limitation of each single comparison study–or as an argument to perform more such comparison studies. Going one step further in the comparison with clinical research, one could also imagine a concept of meta-analysis for comparison studies in computational sciences. In the clinical context, meta-analyses provide a synthesis over different populations, different variants of the investigated therapies, different technical conditions, different medical teams, etc. Similarly, meta-analyses for computational studies in computational sciences would provide syntheses over different data domains, different variants of the considered methods, different software environments, different teams with their own areas of expertise, etc.

### Negative Results and Pitfalls

In our survey none of the 43 papers on new methods presented negative conclusions, suggesting that computational literature is affected by a substantial publication bias. In this context, neutral comparison studies can be a good vehicle for negative research findings. Publication biases and the necessity to “accentuate the negative” [11] are well-documented in the context of medical and pharmaceutical research. In applied statistics and data analysis research, however, this issue receives very poor attention [6], even if the publication of negative results may be extremely useful in many cases.

The systematic exclusion of negative results from publication might in some cases be misleading. For example, imagine that ten teams around the world working on the same specific research question have a similar promising idea that in fact does not work properly for any reason. Eight of the ten teams obtain disappointing results. The ninth team sees a false positive in the sense that they observe significant superiority of the new promising method over existing approaches although it is in fact not better. The tenth team optimizes the method’s characteristics [5] and thus also observes significant superiority. The two latter teams report the superiority of the promising idea in their papers, while the eight other studies with negative results remain unpublished: a typical case of publication bias. This scenario is certainly caricatural, but similar things are likely to happen in practice although in a milder form. Note that it is very difficult to give concrete examples at this stage, since such stories essentially remain unpublished.

Nevertheless, the publication of negative results might entail substantial problems. Most researchers (including ourselves!) probably have more ideas that turn out to be disappointing than ideas that work fine. Try-and-error is an essential component of research. It would thus be impossible (and uninteresting anyway) to publish all negative results. But then, what was promising and what was not promising? What is likely to interest readers and what was just a bad idea that nobody else would have thought of? Obviously this decision that would have to be taken by reviewers and editors is a subjective one. Assessing whether a new method with negative results deserves publication in a separate paper is anything but trivial. With this in mind, we believe that the publication of negative findings within large well-designed comparison studies would be a sensible compromise in order to diffuse negative findings without congesting the literature with negative papers.

Journals would not have to fear for their impact, since good comparison studies are usually highly accessed and cited. Authors would not be urged to make something out of their promising idea on which they have spent a lot of time: a large comparison study would be an alternative to publish important results and share their vast experience on the topic without fishing for significance. And “fishing for significance” would lose part of its attractiveness. Most importantly, readers would be informed about important research activities they would not have heard of otherwise.

Note that “standard practice rules” in computational sciences (for example regarding the choice of method parameters) are often implicitly the result of comparison studies. For instance, a standard parameter value becomes standard because it yields better results than another value. In other words, negative results are often hidden behind standard practice rules - most of them remaining unpublished. Our point is that this process could be made more transparent and more informative for the readers if these negative results were published within extensive comparison studies.

Drawing the comparison with clinical research from the introduction even further, we also think that it may be interesting to publish articles on *pitfalls*. By “pitfall” we mean the inconveniences of a data analysis method such as, e.g., a non-negligible bias, a particularly high variability, or non-convergence of an algorithm in specific cases that may lead to misleading results. In computational literature such research results are often hidden in the middle of articles that are actually devoted to something else. This is in contrast to clinical research, where pitfalls of existing methods (for example an adverse effect of a drug) may be the main object of an article, even if no alternative solution is proposed (for example in form of an alternative drug).

### Conclusion

Neutral comparison studies are in our opinion crucial to make the establishment of standards more objective and to give a chance to methods that are at first view unspectacular and would otherwise be pigeonholed. They are probably not devoted enough attention in the literature, as suggested by our survey that identified only 12 comparison studies out of a total of 55 articles on supervised classification. However, comparison studies and their impact should not be over-interpreted. Firstly, one should not forget that no method is expected to work well with all data sets (the well-known “no free lunch theorem” [4]). Hence, a method that scores well in many comparison studies may do poorly in a specific data set. Comparison studies are not expected to yield an absolute truth applicable to all situations. They are solely useful to determine general trends that may be useful to the community to select a set of standard methods that often perform well.

Secondly, comparison studies are essentially limited because they rely on the specific and sometimes arbitrary choices regarding the study design: the choice of simplifying evaluation criteria that probably do not reflect the complexity of concrete data analysis situations, the choice of method parameters that may substantially impact the relative performance of the considered methods, and last but not least the choice of specific example data sets.

Thirdly, comparison studies are often underpowered in the sense that the number of included data sets is insufficient considering the high variability of performance across data sets. With a few exceptions (see the comparison of machine learning algorithms based on 65 gene expression data sets [12]), comparison studies most often include up to 10 data sets. The median number of considered data sets was only 5 in the 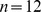 comparison studies considered in our survey. This is probably not enough. This issue may be further investigated in terms of sample size in a statistical testing framework [13].

Fourthly, comparison studies essentially ignore the substantive context of the data sets they consider. Data sets are sometimes preprocessed without much knowledge of the signification of the variables. All methods are applied in the same standardized way to all data sets. The analysis is thus intentionally over-simplified. An important aspect of the data analysis approach is neglected, which does not reflect the complexity and subtleties of the data analyst’s work [14]. A method that does not work well if applied in a standard way without knowledge of the substantive context might perform better in concrete situations, hence reducing the relevance of comparison studies.

To conclude, neutral comparison studies are often considered as less exciting than projects on new methods by both researchers and journal editors–but not by readers. They can neither be expected to always give the best answer to the question “which method should I use to analyze my data set” nor reflect a real data analysis approach that takes the substantive context into account. However, we believe that they may play a crucial role to make the evaluation of existing methods more rational and to establish standards on a scientific basis. They certainly deserve more consideration than is currently the case in the literature.

## Supporting Information

File S1 **Supplementary references.**
(PDF)
