# Supplementary material for: A Plea for Neutral Comparison Studies in Computational Sciences
Source: PLoS One. 2013 Apr 24;8(4):e61562. doi: 10.1371/journal.pone.0061562 (PMC3634809; doi:10.1371/journal.pone.0061562)
Supplement: File S1 — Supplementary references. (PDF) [file pone.0061562.s001.pdf]

Supplementary material

This table lists the references of the papers included in our study and the ID identifying them in the data set `dat_comp.RData`.

Abbreviations of the journal names:

- **BIOINF** – Bioinformatics
- **BMCB** – BMC Bioinformatics
- **CS** – Computational Statistics
- **CSDA** – Computational Statistics and Data Analysis
- **JCGS** – Journal of Computational and Graphical Statistics
- **JMLR** – Journal of Machine Learning Research
- **MACHL** – Machine Learning

**ID**	**Reference**
**BIOINF**	
21_2011_3017_3023	Semi-supervised learning improves gene expression-based prediction of cancer recurrence<br>Shi and Zhang, Bioinformatics 2011 Nov 1;27(21):3017-23
10_2011_1384_1389	High-dimensional pharmacogenetic prediction of a continuous trait using machine learning techniques with application to warfarin dose prediction in African Americans<br>Cosqun et al, Bioinformatics 2011 May 15;27(10):1384-9
01_2011_87_94	Improved performance on high-dimensional survival data by application of Survival-SVM<br>Van Belle et al, Bioinformatics 2011 Jan 1;27(1):87-94

08_2012_1151_1157	Combining multiple approaches for gene microarray classification<br>Nanni et al, Bioinformatics 2012 Apr 15;28(8):1151-7
02_2012_167_175	Feature-based classifiers for somatic mutation detection in tumour-normal paired sequencing data<br>Ding et al, Bioinformatics 2012 Jan 15;28(2):167-75
04_2012_531_537	Improved mean estimation and its application to diagonal discriminant analysis<br>Tong et al, Bioinformatics 2012 Feb 15;28(4):531-7
**BMCB**	
2010_11_427	An adaptive optimal ensemble classifier via bagging and rank aggregation<br>Datta et al, BMC Bioinformatics 2010 Aug 18;11:427
2010_11_523	Class prediction for high-dimensional class-imbalanced data<br>BMC Bioinformatics. 2010 Oct 20;11:523
2011_12_157	Discrimination of approved drugs from experimental drugs by learning methods<br>Tang et al, BMC Bioinformatics 2011 May 14;12:157
2011_12_138	Elastic SCAD as a novel penalization method for SVM classification tasks in high-dimensional data<br>Becker et al, BMC Bioinformatics 2011 May 9;12:138
2011_12_42	Stepwise classification of cancer samples using clinical and molecular data<br>Obulkasim et al, BMC Bioinformatics 2011 Oct 28;12:422
2011_12_153	To aggregate or not to aggregate high-dimensional classifiers<br>Xu et al, BMC Bioinformatics 2011 May 13;12:153
2012_13_139	Comparative study of classification algorithms for immunosignaturing data<br>Kukreja et al, BMC Bioinformatics 2012 Jun 21;13:139
2012_13_178	Finding minimum gene subsets with heuristic breadth-first search algorithm for robust tumor classification<br>Wang et al, BMC Bioinformatics 2012 Jul 25;13:178
2012_13_59	A comparison of feature selection and classification methods in DNA methylation studies<br>using the Illumina Infinium platform<br>Zhuang et al, BMC Bioinformatics 2012 Apr 24;13:59
2011_12_450	Random KNN feature selection - a fast and stable alternative to Random Forests<br>Li et al, BMC Bioinformatics 2011 Nov 18;12:450
**CS**	
2011_26_2_355_36	Classification of repeated measurements data using tree-based ensemble methods<br>Adler et al, Comput. Stat. 2011;26(2):355-369
2012_27_203_218	A boosting method with asymmetric mislabeling probabilities which depend on covariates<br>Hayashi, Comput. Stat. 2012;27(2):203-218
2012_Jan	Biomarker discovery: classification using pooled samples

	Telaar et al, Comput. Stat. 2012;27(1):1-40
2012_May	Partial least squares classification for high dimensional data using the PCOUT algorithm<br>Turkmen et al, Comput. Stat. 2012; DOI:10.1007/s00180-012-0328-y
**CSDA**	
54_2010_1535_1546	Ensemble classification based on generalized additive models<br>De Bock et al, CSDA 2010;54(6):1535-1546
54_2010_1197_1205	The Bayesian Additive Classification Tree applied to credit risk modelling<br>Zhang et al, CSDA 2010;54(5):1197-1205
54_2010_438_451	On the generative-discriminative tradeoff approach: Interpretation, asymptotic efficiency and classification performance<br>Xue et al, CSDA 2010;54(2):438-451
55_2011_1933_1941	Ensemble classification of paired data<br>Adler et al, CSDA 2011;55(5):1933-1941
55_2011_1897_1908	Gene selection and prediction for cancer classification using support vector machines with a reject option<br>Choi et al, CSDA 2011;55(5):1897-1908
56_2012_611_628	A mixed effects least squares support vector machine model for classification of longitudinal data<br>Luts et al, CSDA 2012;56(3):611-628
56_2012_741_751	PCA document reconstruction for email classification<br>Gomez et al, CSDA 2012;56(3):741-751
56_2012_2273_2287	Classification of image pixels based on minimum distance and hypothesis testing<br>Ghimire and Wang, CSDA 2012;56(7):2273-2287
56_2012_1644_1661	Linear discrimination for three-level multivariate data with a separable additive mean vector<br>and a doubly exchangeable covariance structure<br>Leiva and Anuradha, CSDA 2012;56(6):1644-1661
56_2012_2334_2346	Supervised classification for functional data: A weighted distance approach<br>Alonso et al, CSDA 2012;56(7):2334-2346
56_2012_4290_4300	Separable linear discriminant analysis<br>Zhao et al, CSDA 2012;56(12):4290-4300
**JCGS**	
19_1_140-153	Boosting for Correlated Binary Classification<br>Adewale et al, CSDA 2010;19(1):140-153
20_4_901_919	Reinforced Multicategory Support Vector Machines<br>Liu and Ming, CSDA 2011;20(4):901-919
**JMLR**	
11_2010_3183-3234	A Comparison of Optimization Methods and Software for Large-scale L1-regularized Linear Classification

	Yuan et al, JMLR 2010;11:3183-3234
11_2010_491-516	Classification Using Geometric Level Sets<br>Varshney and Willsky, JMLR 2010;11:491-516
11_2010_1353-1390	Learning Translation Invariant Kernels for Classification<br>Ghiasi-Shirazi et al, JMLR 2010;11:1353-1390
11_2010_2901-2934	Linear Algorithms for Online Multitask Classification<br>Cavallanti et al, JMLR 2010;11:2901-2934
11_2010_2199-2228	Regularized Discriminant Analysis, Ridge Regression and Beyond<br>Zhang et al, JMLR 2010;11:2199-2228
11_2010_665-685	Second-Order Bilinear Discriminant Analysis<br>Christoforou et al, JMLR 2010;11:665-685
12_2011_2211-226	Multiple Kernel Learning Algorithms<br>Gonen and Alpaydin, JMLR 2011;12:2211-2268
12_2011_75-110	Multitask Sparsity via Maximum Entropy Discrimination<br>Jebara, JMLR 2011;12:75-110
12_2011_2905-2929	On Equivalence Relationships Between Classification and Ranking Algorithms<br>Ertekin and Rudin, JMLR 2011;12:2905-2929
12_2011_1501-1536	Learning from Partial Labels<br>Cour et al, JMLR 2011;12:1501-1536
13_2012_2107-2143	A Comparison of the Lasso and Marginal Regression<br>Genovese et al, JMLR 2012;13:2107-2143
12_2011_2721-2748	Large Margin Hierarchical Classification with Mutually Exclusive Class Membership<br>Wang et al, JMLR 2011;12:2721-2748
13_2012_607-642	Non-Sparse Multiple Kernel Fisher Discriminant Analysis<br>Yan et al, JMLR 2012;13:607-642
13_2012_1891-1926	Confidence-Weighted Linear Classification for Text Categorization<br>Crammer et al, JMLR 2012;13:1891-1926
13_2012-2279_2292	Pairwise Support Vector Machines and their Application to Large Scale Problems<br>Brunner et al, JMLR 2012;13:2279-2292
**MACHL**	
2010_78_137_17	Methods for the combination of kernel matrices within a support vector framework<br>Martin de Diego et al, Machine Learning 2010;78(1-2):137-174
2010_81_149_178	Learning to classify with missing and corrupted features<br>Dekel et al, Machine Learning 2010;81(2):149-178
2010_80_63_84	Classification with guaranteed probability of error

	Campi, Machine Learning 2010;80(1):63-84
`2011_85_249_272`	Ternary Bradley-Terry model-based decoding for multi-class classification and its extensions<br>Takenouchi and Shin, Machine Learning 2011;85(3):249-272
`2011_85_333_359`	Classifier chains for multi-label classification<br>Read et al, Machine Learning 2011;85(3):333-359
`2012_88_47_68`	Multilabel classification with meta-level features in a learning-to-rank framework<br>Yang and Gopal, Machine Learning 2012;88(1-2):47-68
`2012_88_127_155`	Efficient max-margin multi-label classification with applications to zero-shot learning<br>Hariharan et al, Machine Learning 2012;88(1-2):127-155
`2012_88_5_45`	On label dependence and loss minimization in multi-label classification<br>Dembczynski et al, Machine Learning 2012;88(1-2):5-45
`2012_88_243_272`	Scalable and efficient multi-label classification for evolving data streams<br>Read et al, Machine Learning 2012;88(1-2):243-272
`2012_88_157_208`	Statistical topic models for multi-label document classification<br>Rubin et al, Machine Learning 2012;88(1-2):157-208
`2012_88_209_241`	Synergy of multi-label hierarchical ensembles, data fusion, and cost-sensitive methods for gene functional inference<br>Cesa-Bianchi et al, Machine Learning 2012;88(1-2):209-241
